# Alzheimer’s Disease: Cellular and Pharmacological Aspects

**DOI:** 10.3390/geriatrics9040086

**Published:** 2024-06-22

**Authors:** Gonzalo Emiliano Aranda-Abreu, Fausto Rojas-Durán, María Elena Hernández-Aguilar, Deissy Herrera-Covarrubias, Lizbeth Donají Chí-Castañeda, María Rebeca Toledo-Cárdenas, Jorge Manuel Suárez-Medellín

**Affiliations:** Instituto de Investigaciones Cerebrales, Universidad Veracruzana, Xalapa 91192, Mexico; frojas@uv.mx (F.R.-D.); elenahernandez@uv.mx (M.E.H.-A.); dherrera@uv.mx (D.H.-C.); lchi@uv.mx (L.D.C.-C.); rtoledo@uv.mx (M.R.T.-C.); josuarez@uv.mx (J.M.S.-M.)

**Keywords:** Alzheimer, cell, drugs

## Abstract

Alzheimer’s disease was described more than 100 years ago and despite the fact that several molecules are being tested for its treatment, which are in phase III trials, the disease continues to progress. The main problem is that these molecules function properly in healthy neurons, while neuronal pathology includes plasma membrane disruption, malfunction of various organelles, and hyperphosphorylation of Tau and amyloid plaques. The main objective of this article is the discussion of a neuronal restoration therapy, where molecules designed for the treatment of Alzheimer’s disease would probably be more effective, and the quality of life of people would be better.

## 1. Introduction

Over 100 years ago, Alois Alzheimer described the case of a 55-year-old patient named Auguste Deter. The clinical symptoms she manifested were dementia, with memory loss, delusions, and temporary absence states. She also presented sleep problems; she dragged objects through the house and screamed for hours during the night. After her death, Dr. Alzheimer performed post-mortem studies, where he described for the first time neurofibrillary tangles and plaques in her brain [1]. It is now known that tangles are formed by aggregates of Tau protein and plaques containing amyloid deposits.

Alzheimer’s disease (AD) is the most common form of dementia and one of the major neurodegenerative disorders. It is characterized by a gradual decline in short-term memory and cognitive abilities, which affect the daily functioning of the sufferer.

There are two main types of Alzheimer’s disease: the early-onset form and the late-onset form. Early-onset AD is inherited in a Mendelian dominant manner [2]. Late-onset AD also has a substantial heritable component but involves a wider range of genes in its development. In addition, the progression of late-onset AD is influenced by a combination of genetic and environmental factors. It has been suggested that late-onset AD represents two distinctly separate pathological entities characterized by specific pathophysiological mechanisms attributed to their different genetic bases and uncommon clinical manifestations [3].

Currently, at least 29 genes associated with Alzheimer’s disease have been described through detailed genome-wide association studies (GWAS) [4,5]. In these studies, the gene encoding Tau and the gene encoding the amyloid precursor protein, which by the action of β-secretase 1 and γ-secretase generate amyloid peptides, do not appear as a risk factor due to the post-translational modifications to which these two proteins are subjected after being translated [6,7].

It has been studied in murine models that Tau overexpression causes tauopathy and neurodegeneration [8], and overexpression of the protein precursor β-amyloid induces mitochondrial stress and activates the intrinsic apoptosis cascade [9].

The questions are, how could we treat a disease whose origin is multigenic, and what model would be suitable for a study? We know that one pill will not be able to cure AD. Among the five FDA-approved drugs, donepezil, galantamine, rivastigmine, tacrine, and memantine, unfortunately none of them work, as patients at first seem to recover cognitive ability, but then that ability is lost over time [10].

Therefore, with many genes associated with the disease, what would be the optimal model to study AD?

From our point of view, the only optimal model for this study is the human, but for ethical and sometimes controversial arguments, it is not so easy to do studies in people with the disease.

This only leaves us the option of performing studies in other types of organisms that genetically may have homologies to human beings, and these models may never suffer from AD, since this disease may be limited only to *Homo sapiens*.

Plaques have been found in other organisms with complex brains such as dolphins, where their average lifespan is approximately 58 years [11]. This would be a promising model to study the disease; however, it seems that these organisms, due to their diet and average lifespan, do not present something similar enough to the disease.

The following is a narrative review where a literature search and review was performed in PubMed on the study models in Alzheimer’s disease, as well as the main molecules that are in phase III clinical trials for the possible treatment of this neurodegenerative disease. Keywords such as Alzheimer’s models, molecules, phase III, and clinical trials were used.

## 2. Models for the Study of AD

There are several study models [12,13], from cell cultures to genetically modified organisms. Regarding animal models, there is the 3XTg-AD mouse model, which has mutations associated with familial Alzheimer’s disease (APP Swedish, MAPT P301L and PSEN1 M146V). It presents plaques and tangle pathology in the hippocampus and cerebral cortex [14]. The 5XFAD mouse model expresses human APP and PSEN1 transgenes with a total of five mutations linked to Alzheimer’s disease: the Swedish (K670N/M671L), Florida (I716V), and London (V717I) mutations in APP and the M146L and L286V in PSEN1, showing amyloid plaques with gliosis, neuronal loss in several brain regions as well as cognitive and motor deficits [15]. There is an extensive list of animal models for the study of Alzheimer’s disease (https://www.alzforum.org/research-models/alzheimers-disease, accessed on 20 April 2024.).

Other models for the study of the disease are cell cultures, such as the use of iPSC cells (induced pluripotent stem cell-derived neurons), where mutations of the PSEN1 and PSEN2 genes as well as APP and Tau phosphorylation have been investigated [16]. Primary cultures of microglia isolated from human and transgenic mouse brains have been used to study Tau spreading in the brain [17]. Co-culture of PC12 cells, together with C6 glioma cells, has been used as a model of sporadic Alzheimer’s disease, where APP and β-secretase have been assayed when there is a decrease in insulin receptor expression [18]. Culture studies in 3D have also been carried out, as well as brain organoid cultures, to try to accelerate the pathology of AD [19]. Yeast models have been used to study Tau and β-amyloid aggregation [20]. Other cell lines also used in the study of AD are HEK 293 cells, [21], SH-SY5Y [22], P19 [23,24,25], and PC12 [26].

The zebrafish model has been implemented for the study of AD, as it could be used as a behavioral/pharmacological model or both [27]. *Drosophila* has emerged as a good model to study AD, as these organisms present genotypes similar to AD [28].

The various studies in in vitro models as well as laboratory animals have led to the development of drugs that could mitigate the effects of the disease.

## 3. Phase III Drugs for AD

### 3.1. Antibodies

The recently approved lecanemab is able to bind with high affinity to β-amyloid protofibrils and has been able to modestly improve measures of cognition in patients 18 months after use relative to those receiving a placebo. This antibody is administered intravenously at a dose of 10 mg/kg body weight every two weeks [29].

Solanezumab, a monoclonal antibody designed to eliminate soluble amyloid peptides that can generate toxic effects in the synapse and precede the deposition of amyloid fibers, showed no significant effect in improving cognitive impairment in a total of 578 patients with moderate Alzheimer’s disease. It was applied at a dose of 1600 mg intravenously every 4 weeks for 240 weeks [30,31].

Aducanumab, an antibody capable of reducing plaques Aβ in patients with early disease, appears to lead to slight improvement, as shown in studies with MMSE. The dose used was 10 mg/kg intravenously for two months, with initial doses of 1 mg/kg to 3 mg/kg [32,33].

Gantenerumab, an anti-Aβ monoclonal antibody that has been used in patients with moderate Alzheimer’s disease, showed a slight decrease in CFS when administered at doses of 105 mg and 225 mg every 4 weeks by subcutaneous injection for 2 years of treatment [34,35].

Crenezumab, a monoclonal antibody designed to remove toxic beta-amyloid oligomers, has not been successful in Alzheimer’s patients until now [36]. It was applied at a dose of 60 mg/kg intravenously every 4 weeks for 100 weeks [37].

### 3.2. Inhibitory Molecules

Verubecestat (MK-8931) is a BACE1 inhibitor that was administered to patients with AD onset at different doses of 12 and 40 mg per day. Results showed that verubecestat did not improve clinical assessments of dementia among patients with prodromal AD, and some measures suggested that cognition and daily function were worse among patients who received verubecestat than among those who received a placebo [38].

Lanabecestat (AZD3293) is a potent BACE1 inhibitor that, at different doses from 20 to 50 mg for 104 weeks, was not shown to reduce Aβ levels in CFS [39].

ALZ-OP1 is a combination treatment of cromolyn sodium, which is a mast cell stabilizer used for the treatment of asthma that acts as an anti-inflammatory to inhibit the release of cytokines, and ibuprofen, an NSAID that is a selective COX inhibitor that can reduce Aβ_1-42_ peptide levels by modulating the activity of β-secretase. The results of this compound are in the results phase [40].

Azeliragon is a receptor for advanced glycation endproducts (RAGE) inhibitor. Studies have shown that it can reduce amyloid plaques, and studies in Alzheimer’s patients at a dose of 5 mg/day have shown that treatment for 18 months improves mild to moderate cognitive impairment [41].

TRx0237 is a small molecule that inhibits Tau aggregation; it is being applied to patients with mild Alzheimer’s disease, and the results of this treatment are still being awaited [42] from clinical trial NCT03446001 [43].

The amyloid peptide aggregation inhibitor elenbecestat is currently in phase III studies. The results of the application of this inhibitor in CSF are still being awaited; the doses being studied range from 5 to 50 mg/day [44].

GV-971 is a novel oligosaccharide that targets amyloid fiber formation and neuroinflammation. The results have shown an improvement in the cognitive capacity of patients; therefore, this molecule is emerging as a beneficial treatment for patients with Alzheimer’s disease (Table 1) [45].

### 3.3. Molecules That Reduce the Symptoms of the Disease

Various drugs that can reduce the symptoms of AD have been tested, including escitalopram (serotonin reuptake inhibitors), methylphenidate (central nervous system stimulant), nabilone (cannabinoid agonist), zolpidem (benzodiazepine analogue), and suvorexant (orexin receptor antagonists) [46].

It is important to note that type III diabetes has been described as being due to insulin resistance within the brain [54]. Consequently, there are several drugs that influence glucose metabolism under clinical investigation in phase 3. A multicenter phase 2/3 prevention trial began in March 2021. Metformin was used in the Alzheimer’s Dementia Prevention (MAP) study that enrolled 326 people at more than 20 academic medical centers in the U.S. The participants were people between 55 and 90 years of age, overweight or obese without diabetes, and having early or late mild cognitive impairment [55]. Insulin has been shown to modulate several aspects of brain function relevant to Alzheimer’s disease and can be delivered to the brain by intranasal devices [56,57].

However, despite the efforts that have been made to find a suitable drug that could at least stop the disease, currently nothing has yet been found.

Another question is, what are we doing wrong? It has been seen that models provide us important information about how the disease may originate; however, these models, like any other model in biology, have their limitations.

The drugs that have been developed are undoubtedly well designed to meet their objectives, but why do they not work? Is it because they are designed for cells that are not damaged? Perhaps this applies to neurons that will never show a phenotype like AD, as is the case of the models that are used today. This is why it is important to take into account cellular functioning in order to design molecules that can repair the damage that is caused intracellularly by Alzheimer’s disease.

## 4. Back to Basic Cell Biology

Every cell is surrounded by a phospholipid membrane that allows it to maintain its cellular contents, i.e., cytoplasm and organelles, which also have membranes to protect their own molecular contents.

Studies have shown that amyloid peptide oligomers are capable of destroying membrane integrity and cellular homeostasis [58]. It has been determined that the endocytic pathway could be affected, and therefore affect the traffic of proteins such as APP and BACE1, since proteins that are part of the endocytic pathway could be compromised, such as RAB7a, sortin nexin 3, and sortilin protein-related receptor, among others [59]. It has also been determined that the nuclear membrane is affected by AD by reducing the expression levels of B-type lamin protein due to the overexpression of Tau [60]. The mitochondrial membrane may be affected by the action of amyloid and Tau peptides, resulting in mitochondrial dysfunction causing primarily bioenergetic and respiratory chain failure [61]; moreover, mitochondria associated with the endoplasmic reticulum may also be affected [62].

These are some cases where membranes are compromised due to AD, since the overexpression of Tau or its hyperphosphorylation, as well as the presence of amyloid peptides, may be responsible for a generalized failure at the cellular level; the endocytic pathway is affected, and the receptors involved in neurotransmission will also be damaged, either in their tertiary structure or in their intracellular transit [63], which could indicate that the rough endoplasmic reticulum is under stress [64]. It is important to mention that, as the cell membranes are affected, neurotransmission will not be effective; for example, to form the neurotransmitter acetylcholine, acetyl-CoA from the mitochondria and choline are required to form acetylcholine by the enzyme acetyltransferase [65].

As mentioned, as the mitochondria are affected, the synthesis of this neurotransmitter is compromised in patients with Alzheimer’s disease. In addition, cholinergic receptors may be disrupted as they cannot be properly transported to their specific site in the membrane. The same may occur with glutamate receptors and acetylcholine [66,67].

Another very important part to be addressed is the stress response of the endoplasmic reticulum; this is a response to the imbalance in its homeostasis where transmembrane proteins and those that will be secreted are assembled. This imbalance corresponds to various factors such as the accumulation of misfolded proteins, alterations in the redox balance, protein overload, or viral infections.

The proteins that are involved in the stress response include IRE 1 (inositol-requiring enzyme 1), which is activated by the accumulation of misfolded proteins. Upon activation, IRE1 will cleave XBP1 (X-box binding protein 1) pre-mRNA, generating a spliced form of XBP1s. XBP1s acts as a transcription factor that induces the expression of genes involved in the RER stress response, such as the chaperone GRP78, and degradation proteins such as ERAD (ER-associated degradation) and UPR (unfolded protein response) proteins [68].

Another protein is PERK (PRKR-like endoplasmic reticulum kinase), which is also activated by misfolded proteins. When activated, PERK phosphorylates eIF2α (eukaryotic initiation factor 2 alpha), which reduces protein translation globally. PERK also induces the expression of genes involved in the RER stress response [69].

Another protein involved in this stressful process is ATF6 (activating transcription factor 6), a transmembrane protein that is activated by proteolysis in the RER. The released portion of ATF6 translocates to the nucleus where it acts as a transcription factor, regulating genes involved in the RER stress response [70].

ATF4 (activating transcription factor 4) is a transcription factor that is activated by phosphorylation of eIF2α by PERK. ATF4 induces the expression of genes involved in the RER stress response such as chaperones and UPR proteins [71].

CHOP (C/EBP homologous protein) is a transcription factor that is induced by the activation of PERK and IRE1. CHOP protein induces the expression of genes involved in apoptosis in the case of persistent stress [72].

Drugs designed to prevent RER stress are being developed that target the accumulation of misfolded proteins such as protease inhibitors, protein misfolding correctors, and UPR enhancers. Drugs that are PERK and IRE1 inhibitors, as well as ATF6 modulators, are also being developed. Drugs targeting Ca^2+^ modulation as well as channel modulation are also being developed.

## 5. Drugs under Development for RER Stress

Velcade (bortezomib) is a drug used primarily to treat multiple myeloma, but because it is a proteosome inhibitor, it may be useful in inhibiting RER stress by reducing the accumulation of misfolded proteins. However, because it has serious side effects such as fatigue, peripheral neuropathy, and gastrointestinal problems, it has not been recommended for use in RER stress [47].

Carfilzomib, a second-generation proteosome inhibitor, is used in the treatment of multiple myeloma. This drug acts by inhibiting the proteosome, which may help to improve RER function [48].

VX-809, known as lumacaftor, is an approved drug for cystic fibrosis; however, recent research has suggested it as a treatment for RER. VX-809 acts as a folding corrector in the case of the protein encoded by the CFTR gene, allowing it to reach the plasma membrane and improve its function [49].

ISRIB is a small molecule that restores the translation of eIF2α, conferring neuroprotection. ISRIB can partially restore the rate of protein synthesis for neuron survival [50].

Salubrinal, an experimental drug that has been investigated as a treatment for RER stress, acts by inhibiting the eIF2α [51].

GSK2606414 is a selective protein kinase R (PERK) inhibitor drug, which could help alleviate stress [52].

Ceapin A, a natural compound found in the Chaga mushroom (Inonotus obliquus), may act by modulating the activity of the transcription factor ATF6 [53].

Therefore, the drugs that have been approved for the treatment of AD have not been effective, not because they are poorly designed, but because metabolic and cellular functions are affected, and they will not correct the damage but maximize neuronal function: on the one hand, by inhibiting acetylcholinesterase and, on the other hand, by exerting their antagonistic action on glutamate receptors, as is the case of memantine.

**Figure 1:**
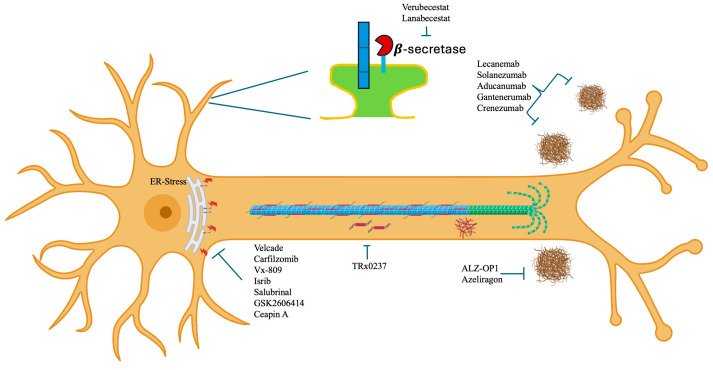
Phase III drugs and molecules being tested for the treatment of Alzheimer’s disease applied at different sites in the neuronal cytoplasm.

The main problem lies in the fact that AD, being multigenic, affects many metabolic pathways and neuronal functions that cannot be resolved with a pill or an antibody (Figure 1).

## 6. What Do We Do?

One of the important aspects is that both the person and family members are made aware of the warning signs that may appear, which indicate that the disease has already advanced; however, a treatment called “neuronal rehabilitation” could be started, that is, trying to repair the damage of the cells at the membrane level and reduce the neuroinflammatory process.

## 7. Neural Rehabilitation Process

An important aspect that needs to be resolved first is neuroinflammation, where cytotoxicity and an inflammatory response that includes the protein β-amyloid, cytokines, and prostaglandins that generate cyclooxygenase type 2 (COX-2) have been determined to be involved [73].

A successful way to diminish neuroinflammation is to use NSAIDs, which are capable of decreasing the levels of COX-2 expression. Celecoxib and nimesulide have been the most commonly used NSAIDs. In clinical trials, celecoxib has not appropriately decreased the levels of the COX-2 enzyme [74]; however, nimesulide has been effective, reducing the levels of prostaglandins and COX-2 [75].

On the other hand, memory loss represents one of the most severe symptoms of AD, as the person begins to forget, little by little, and gradually loses the details of daily life to the point of forgetting who they are.

Neuroinflammation is of great importance in AD, since neuroinflammation has been identified as a key trigger of the disease [76,77]. NSAIDs have a protective effect in rodents treated with quincalic acid injected into the nucleus basalis; excitotoxin induced cholinergic degeneration and intense glia reaction as well as inflammatory mediator production. In rodents treated with nimesulide (10 mg/kg/day, i.m.), microglia reaction was strongly attenuated, along with decreased nitric oxide synthase activity and completely inhibited prostaglandin-E2 formation [78].

Nimesulide is an NSAID that specifically suppresses COX-2, which could be beneficial in the treatment of AD [79] caused by the neuroinflammation process typical of this pathology, which involves the increase in activated microglia and astrocytes. As well, some T cells, along with a molecular alteration characteristic of inflammation such as cytokines, are partially triggered by the accumulation and extracellular precipitation of neurofibrillary tangles [80]. Epidemiological studies of people who took nonsteroidal anti-inflammatory drugs (NSAIDs) have observed a delay in the onset of AD, for instance in identical twins who received anti-inflammatory treatment related to the damage caused by neuroinflammation [81]. Early studies suggest that a potential mechanism of action of NSAIDs is the modulation of gamma secretase activity, which is required for the proteolytic anchoring of APP and the production of β-amyloid peptide [82].

The prescribed dose of 100 mg per day is advised based on the pharmacokinetics of nimesulide, as it leads to plasma concentrations reaching up to 4.58 mg/L in approximately 3 h [83].

The recommended administration is for only one month due to the risk of hepatotoxicity [84].

The pharmacological data indicate that citalopram, a selective serotonin reuptake inhibitor, is associated with cholinergic modulation of monoamines in Alzheimer’s disease (AD). Investigation of this drug is focused primarily on evaluating its efficacy in ameliorating agitation in people with Alzheimer’s disease [85]. Additionally, citalopram has been reported to have an effect on Tau hyperphosphorylation in the rat hippocampus. Within this experiment, rodents were placed in social isolation; it was reported that the enhanced level of phosphorylated Tau observed in social isolation can be reverted by citalopram [86]. Its effects on Tau acetylation or its hyperphosphorylation under the action of β-amyloid peptide are unknown. The recommended dose to enable the drug to maintain its effect in elderly patients is 20 mg per day [87].

Resveratrol, a well-known polyphenol with cardioprotective properties in the field of cardiovascular diseases and cancer, is also involved in the regulation of pathological processes associated with diseases such as stroke, ischemia, and Huntington’s disease [88]. Numerous studies indicate the therapeutic efficacy of the treatment. Studies have shown a reduction in β-amyloid peptide generation and a possible downregulation of Tau hyperphosphorylation, in conjunction with the abnormal aggregation observed in animal models [89]; nevertheless, it is still uncertain which effect it has on the Tau protein. The proposed mechanism of action could be through the activation of SIRT1, which is a deacetylase with antioxidant effects. Deacetylation of the Tau protein prevents the destabilization of microtubules and reduces the formation of neurofibrillary tangles, inhibiting p53; therefore, apoptosis is reduced, reducing the inflammatory process and neurotoxicity, increasing the activity of alpha-secretase, and decreasing the yield of β-amyloid peptide [90].

A dose of 75 mg per day has been established as adequate for the prevention of microvascular dysfunction in individuals diagnosed with type II diabetes [91], where this condition has been identified as a possible determinant factor for the onset of AD [92].

To preserve the well-being of neurons, it is essential to maintain the structural integrity of the plasma membrane and to mitigate the impact of oxidative stress [93]. Attention has been directed towards omega-3 fatty acids, which are essential for the correct maintenance of the plasma membrane [94,95]. Omega-3 fatty acids have been shown to maintain brain structure and function, suggesting a possible role in postponing dementia [96].

A dose of 800 mg of omega-3 acids has been recommended to decrease the rate of dementia in older adults [97].

**Figure 2:**
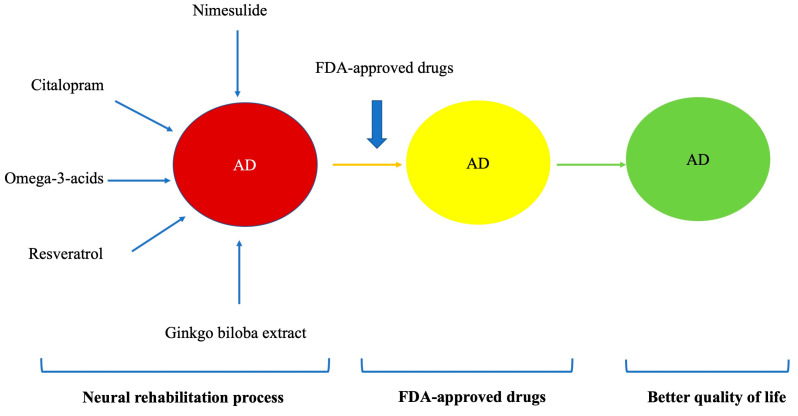
Different phases of the neurorehabilitation process.

The extract of Ginkgo biloba has been used for the management of memory problems [98,99] with good results in cognitive function. Research has determined that a daily dose of 240 mg of ginkgo biloba extract Egb 761 is effective in increasing prefrontal dopamine [100], which is the dose that can be safely taken [101] (Figure 2).

## 8. Conclusions

One drug will not be enough to cure Alzheimer’s disease; we are of the opinion that neuronal restoration therapy must be carried out first before FDA-approved drugs are used.

Synthesized drugs will not be effective unless neuronal membranes are functioning properly.

Biological membranes must be restored, with decreased phosphorylation of Tau and amyloid plaques, making neuronal connections, and decreased oxidation; after that, any drug or molecule that is designed can be more effective on the disease.

Neuronal rehabilitation therapy will allow the neurons that are still viable to recover so that the approved treatments can act efficiently, allowing the functional recovery of the neurons. For this reason, it is important that cell metabolism be restored, in order to have better neuronal functioning and for the approved drugs to exert their effects on cells in an efficient way.

Although this article focused mainly on cellular and pharmacological aspects, it is also important to mention that along with the neuronal restoration process, a balanced diet containing antioxidants should be followed to minimize reactive oxygen species as much as possible, which are harmful to biological membranes. It is also important that the patient leads an active life, i.e., that he/she is not isolated but always part of the family circle, and that his/her brain is constantly stimulated.

## Figures and Tables

**Table 1:** Molecules used against Alzheimer’s disease.

*Molecule*	Dose	Effect	Reference
*Lecanemab*	10 mg/kg, IV	In amyloid plaques.	[29]
*Solanezumab*	1600 mg, IV	Aβ-peptide, soluble monomers, no extracellular plaques.	[31]
*Aducanumab*	10 mg/kg, IV	N-terminal of the amyloid peptide.	[33]
*Gantenerumab*	225 mg, subcutaneous	Amyloid plaques.	[35]
*Crenezumab*	60 mg/kg, IV	It binds monomeric and aggregated Aβ, with higher affinity for oligomeric Aβ.	[37]
*Verubecestat*	12 and 40 mg per day	Inhibitor of β-secretase 1.	[38]
*Lanabecestat*	20 and 50 mg	Inhibitor of β-secretase 1.	[39]
*ALZ-OP1*	Inhaled in combination with ibuprofen	Reduces aggregates from Aβ.	[44]
*Azeliragon*	5 mg/day	The deposition of amyloid plaques decreases.	[41]
*TRx0237*	In progress	Inhibits Tau aggregation.	[43]
*Elenbecestat*	Phase III	Inhibits aggregation of amyloid peptides.	[45]
*GV-971*	In progress	Oligosaccharide against the formation of amyloid fibers and neuroinflammation.	[45]
*Escitalopram*	In progress	Serotonin reuptake inhibitor.	[46]
*Methylphenidate*	In progress	Central nervous system stimulant.	[46]
*Nabilone*	In progress	Cannabinoid agonist.	[46]
*Zolpidem*	In progress	Benzodiazepine analog.	[46]
*Suvorexant*	In progress	Orexin receptor antagonist.	[46]
*Velcade*	RER use is suggested	Proteosome inhibitor, could be used in RER stress.	[47]
*Carfilzomib*	Suggested use RER	Proteosome inhibitor.	[48]
*VX-809*	Suggested use RER	Corrects protein folding.	[49]
*ISRIB*	Use in RER	Restores protein synthesis.	[50]
*Salubrinal*	Stress in RER	Acts by inhibiting eIF2α	[51]
*GSK2606414*	Stress in RER	Selectively inhibits protein kinase R.	[52]
*Ceapin A*	Stress in RER	It acts by modulating the activity of the transcription factor ATF6.	[53]

## Data Availability

No new data were created or analyzed in this study. Data sharing is not applicable to this article.
